# Small Semantic Networks in Individuals with Autism Spectrum Disorder Without Intellectual Impairment: A Verbal Fluency Approach

**DOI:** 10.1007/s10803-020-04457-9

**Published:** 2020-03-20

**Authors:** Felicitas Ehlen, Stefan Roepke, Fabian Klostermann, Irina Baskow, Pia Geise, Cyril Belica, Hannes Ole Tiedt, Behnoush Behnia

**Affiliations:** 1 Present Address: Department of Neurology, Motor and Cognition Group, Charité – Universitätsmedizin Berlin, Campus Benjamin Franklin (CBF), Hindenburgdamm 30, 12203 Berlin, Germany; 2 Present Address: Department of Psychiatry, Jüdisches Krankenhaus Berlin, Heinz-Galinski-Straße 1, 13347 Berlin, Germany; 3 Department of Psychiatry, Charité – Universitätsmedizin Berlin, Campus Benjamin Franklin (CBF), Hindenburgdamm 30, 12203 Berlin, Germany; 4 Berlin School of Mind and Brain, Humboldt-Universität Zu Berlin, Unter den Linden 6, 10099 Berlin, Germany; 5 Department of Psychology, Humboldt-Universität Zu Berlin, Unter den Linden 6, 10099 Berlin, Germany; 6 Present Address: Universität Potsdam, Am Neuen Palais 10, 14469 Potsdam, Germany; 7 Department of Digital Linguistics, Leibniz-Institut für Deutsche Sprache, R5, 6-13, 68161 Mannheim, Germany

**Keywords:** ASD, Verbal fluency, Mental lexicon, Clusters, WCC

## Abstract

Individuals with Autism Spectrum Disorder (ASD) experience a variety of symptoms sometimes including atypicalities in language use. The study explored differences in semantic network organisation of adults with ASD without intellectual impairment. We assessed clusters and switches in verbal fluency tasks (‘animals’, ‘human feature’, ‘verbs’, ‘r-words’) via curve fitting in combination with corpus-driven analysis of semantic relatedness and evaluated socio-emotional and motor action related content. Compared to participants without ASD (n = 39), participants with ASD (n = 32) tended to produce smaller clusters, longer switches, and fewer words in semantic conditions (no *p* values survived Bonferroni-correction), whereas relatedness and content were similar. In ASD, semantic networks underlying cluster formation appeared comparably small without affecting strength of associations or content.

## Introduction

Autism Spectrum Disorder (ASD) is understood as a group of neurodevelopmental disorders characterised by difficulties in social interaction and communication as well as stereotyped patterns of behaviour or restricted interests. Clinical presentation and individual symptom severity are highly variable and range from circumscribed atypicalities with normal or superior intelligence to severe impairments in daily living which can be accompanied by intellectual impairments (American Psychiatric Association 2013; Masi et al. 2017). Difficulties in language (Groen et al. 2008) and motor functions (Fournier et al. 2010) are commonly associated with ASD. During the last decades, prevalence has increased and is currently estimated to be up to 1 in 59 children with a predominance in males (with males being four times more likely to be affected; Baio et al. 2018). While its aetiology is only partially understood, multiple genetic and epigenetic deviations have been identified and are expected to interact with environmental factors (Wiśniowiecka-Kowalnik and Nowakowska 2019). Influential cognitive models tackling the core features of ASD provide evidence for, e.g. executive dysfunction (e.g. Ozonoff 1997), weak central coherence (WCC; Frith 1989), and impaired theory of mind (ToM; Baron-Cohen et al. 1985), each explaining certain but not all of the symptoms associated with ASD and possibly acting in combination (Happé and Frith 2006). In this sense, impaired executive functions, including mental flexibility, have been proposed to account for rigid, repetitive, and maladaptive behaviours in individuals with ASD (Ozonoff 1997). The WCC theory proposes a bias towards local over global processing in ASD which may explain superior performance in detail-focussed processing at the expense of generalisation (for a review see Happé and Frith 2006). From a neurofunctional perspective, this bias has been related to a functional underconnectivity of integrative networks in individuals with ASD (Just et al. 2004) and a growing number of neuroimaging studies provide support for aberrant (e.g. Arnold Anteraper et al. 2018; Belmonte 2004; Catani et al. 2016; Ecker et al. 2016; Hong et al. 2019), mainly reduced, connectivity (for a review see Hull et al. 2017) of the involved cerebral networks. ToM captures the idea of being able to perceive social cues, and to derive and interpret other people’s mental states, beliefs, and thus intentions (Premack and Woodruff 1978). ToM deficits in persons with ASD have therefore been associated with challenges in social interaction, e.g. regarding the prediction of the other’s emotions and actions (Baron-Cohen et al. 1985).

In addition to their implications for social interaction, all three cognitive theories provide explanations for some of the language difficulties described for persons with ASD and shall be outlined below (for reviews see Groen et al. 2008; Martin and McDonald 2003). Clinically, the majority of persons with ASD experience some degree of impaired use and perception of semantics, syntax, pragmatics, i.e. how language is used in a social context (Eales 1993; Fine et al. 2011), and sometimes phonology (Tager-Flusberg et al. 2005; for a review see Groen et al. 2008). The clinical spectrum, however, ranges from a complete inability to acquire speech in a minority of individuals to subtle atypicalities mainly regarding pragmatics in individuals with ASD without intellectual impairment (Kjelgaard and Tager-Flusberg 2001; Tager-Flusberg et al. 2005). Corresponding peculiarities involve the use of less ‘typical’ words and have therefore been proposed to originate from an atypical structure of lexical associations (Dunn et al. 1996).

Theories on the neural underpinnings of the lexical system have become increasingly complex (e.g. Binder and Desai 2011; Patterson et al. 2007; Rofes et al. 2019) and while a great amount of knowledge has been gained, many issues are still unanswered. There has been a general move away from regionally and functionally circumscribed language areas towards a widely distributed, multi-modal network (e.g. Binder and Desai 2011; Kiefer and Pulvermüller 2012; Labache et al. 2019; Mirman et al. 2015; Patterson et al. 2007; Tremblay and Steven 2017). Separable sub-systems are suggested to serve language production vs. recognition (Mirman et al. 2015), semantic vs. phonologic processing (Hickok and Poeppel 2004; Mirman et al. 2015) as well as syntactic (Rofes et al. 2019) and orthographic (Peleg et al. 2016; Seidenberg and McClelland 1989) processing. In terms of language production, neurolinguistic models have thus suggested multi-level processing including conceptualisation, lexical-semantic access, syntactic and phonological encoding, and articulatory preparation (e.g. Dell 1986; Indefrey and Levelt 2004; Levelt 1999; Walker and Hickok 2016; for a review, see Henry and Crawford 2005) leaving the debate open as to whether the last steps are achieved sequentially (e.g. Indefrey and Levelt 2004; Roelofs et al. 1996; Levelt 1999) or in an interconnected mode (e.g. Dell 1986). There is, however, consensus on the idea of an initial conceptualisation, i.e. the activation of non-verbal representations of an object’s sensory, motor, and affective features (encompassing, e.g. shape, use, familiarity, and relationships with other objects; Binder and Desai 2011; Kiefer and Pulvermüller 2012; Levelt 1999; Rofes et al. 2019; Pulvermüller 1999). These featural representations have been proposed to be “stored in semantic memory” (Binder and Desai 2011) in a fashion that modality specific information is connected (Patterson et al. 2007) or gradually converges to form more abstract concepts eventually allowing for lexical-semantic operations (Binder and Desai 2011; Damasio et al. 1994; Rofes et al. 2019). This network structure could account for semantic associations (Rofes et al. 2019) between distinct items which thus share common “conceptual memory traces” (Kiefer and Pulvermüller 2012), including perceptual features (e.g. the feature ‘furry’ could be shared by ‘cat’ and ‘rabbit’) as well as individual values (e.g. the feature ‘safe’ could be shared by ‘work’ and ‘home’; cf. Murphy and Medin 1985). Within this system, prefrontal functions should enable access to, selection of, and shifting between the thus stored semantic knowledge (Mirman et al. 2015; Binder and Desai 2011).

Against this background, executive dysfunction in individuals with ASD could entail a less flexible shift away from one focus to another (Kleinhans et al. 2005; Ozonoff et al. 2007) thus impeding a swift flow of language (Martin and McDonald 2003). Impaired ToM, on the other hand, may account for pragmatic difficulties in individuals with ASD via reduced meta-representational capacities (Baron-Cohen 1988), such as abstraction and presupposition (Boucher 2003; Eales 1993; Helen Tager-Flusberg and Sullivan 1995; Martin and McDonald 2003). Moreover, a crucial overlap between brain regions involved in social cognition and the semantic network has been proposed as a neural connection between impaired ToM and semantic difficulties (Binder and Desai 2011; Groen et al. 2008). From the viewpoint of WCC, semantic deviations in persons with ASD could be theorised as sequalae of weak integrative functions within the semantic network (Huemer and Mann 2010; Nation 1999; Happé and Frith 2006). Likewise, pragmatic deviations have been discussed as reduced contextual integration of the social features of an utterance (Happé and Frith 2006; Martin and McDonald 2003). Moreover, underconnectivity between frontal and parietal areas in individuals with ASD during sentence comprehension first suggested an association between WCC and functional hypoconnectivity within the language system (Just et al. 2004). Specific implications for language production have been indicated by functional connectivity and tractography studies suggesting a general (Verly et al. 2014) as well as region specific (Verly et al. 2014; Knaus et al. 2010) hypoconnectivity involving various frontal and temporal language regions during word production tasks.

From the above considerations on how mental representations presumably shape the semantic network, it appears plausible that difficulties in social interaction could particularly affect language use involving social interactions or emotional references (Binder and Desai 2011). Corresponding clinical studies, however, yielded controversial results indicating both impaired (Begeer et al. 2010; Brown et al. 2012) and unaffected (Bang et al. 2013; Helen Tager-Flusberg and Sullivan 1995) socio-emotional language use.

Similarly, hypothesising an embedding of the semantic network in the sensorimotor system (e.g. Kiefer and Pulvermüller 2012; Pulvermüller 1999; Rofes et al. 2019), a vulnerability of motor action related language to motor impairments has been proposed (Neininger and Pulvermüller 2001 cf. Aravena et al. 2014). Considering the high prevalence of motor symptoms in individuals with ASD (for a meta-analysis see Fournier et al. 2010), particular difficulties in the production of motor action words could thus be expected.

Against this theoretical background, the present study aimed to assess group differences in Verbal Fluency (VF) tasks that could allow conclusions on semantic network organisation, executive functions, or content specific peculiarities in adults with ASD without intellectual impairment vs. non-ASD.

During VF tasks, participants are asked to produce as many words as possible belonging to a predefined semantic category within a limited amount of time (Bousfield and Sedgewick 1944). In so doing, speakers will naturally alternate between rapid production of closely associated words (i.e. ‘clusters’) and pauses between less connected words (i.e. ‘switches’) (Gruenewald and Lockhead 1980). Whereas the former is thought to result from an automatic spreading activation between closely related items (Collins and Loftus 1975; Roelofs 1992), the ability to switch is seen as a frontal executive function enabling strategic retrieval (Troyer et al. 1997; Turner 1999). Constraining the VF condition to a particular initial letter (i.e. ‘letter fluency’) instead of a semantic category (i.e. ‘semantic fluency’) requires a search strategy based on orthographic properties (Birn et al. 2010), while suppressing automatic but inappropriate semantic co-activation (Robinson et al. 2012; Vonberg et al. 2014).

**Fig. 1:**
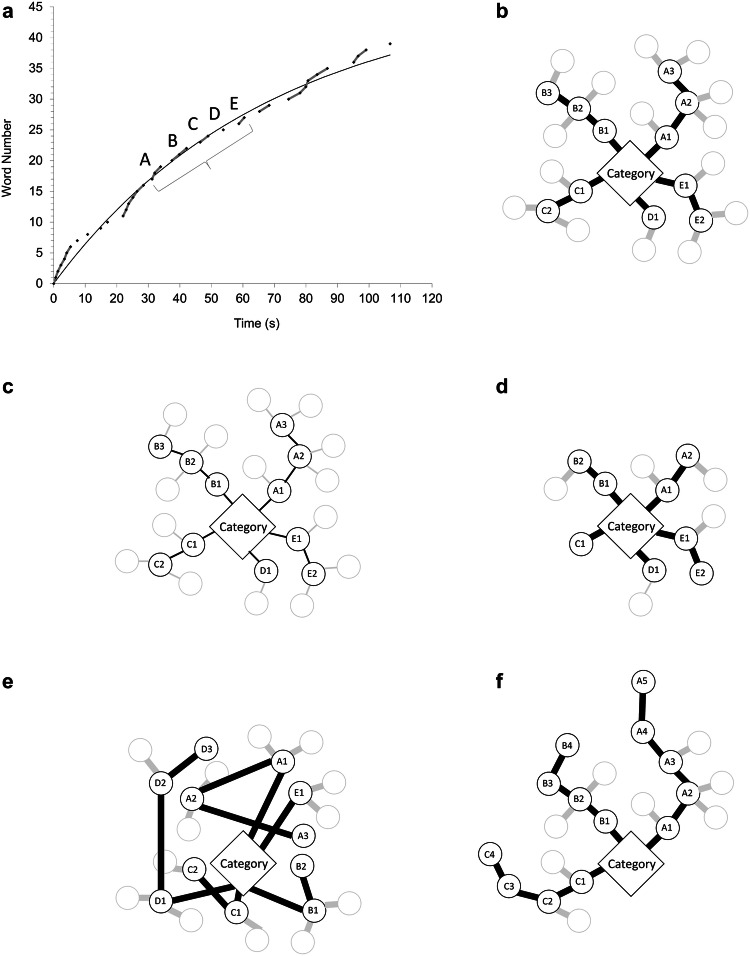
Semantic Network Model. **a, b** Model of typical network function: By means of curve fitting, consecutive words (word number on the ordinate) are plotted as a function of time (starting time in seconds on the abscissa). Applying a slope difference algorithm, temporal clusters (e.g. clusters A, B, C, D, E) with a faster production rate can be differentiated from slower switches. As exemplified by clusters A–E, word retrieval can be understood as an activation of the task category (rhombus) followed by an automatic activation of the first cluster of closely related words (A1–A2–A3). Noteworthy, due to task constraints the number of uttered words (black circles) does not necessarily represent the number of all mental associations (black + grey circles). If, e.g. the words ‘pig-horse-cow’ were correctly retrieved as belonging to the category ‘animals’, the association ‘stable’ would have to be suppressed. Once a cluster has ended, a switch will occur during which a new word will be actively searched, leading to either the activation of another cluster (B1–B2–B3) or another switch (D1). ‘Cluster size’ (i.e. number of words per cluster) is thus a marker for the scope of densely related words; ‘intracluster time’ (i.e. intervals between consecutive words within the same cluster) a marker for strength of established associations between lexical items; ‘number of switches’ a marker for accessible items in a given amount of time, and ‘switch duration’ (i.e. interval between two words not belonging to the same cluster) a marker for accessibility. Semantic relatedness (i.e. co-occurrence value) should be related to the typicality of semantic associations. **c** Model of weaker semantic associations: Due to less efficient automatic activation, longer intracluster times with unaltered cluster size should be expected. Consequently, fewer switches within the given amount time should occur. Due to a preserved overall organisation of associations, semantic relatedness should not be affected. **d** Model of fewer densely connected words: Smaller cluster sizes should represent a lower number of highly associated words and go along with unaltered intracluster time, switch duration, and semantic relatedness. **e** Model of atypical associative pattern: Deviations from the typical organisation of semantic associations should lead to a lower semantic relatedness. **f** Model of impaired set shifting function: Reduced set shifting as an executive dysfunction should lead to longer attachment to a cluster rendering larger clusters, fewer switches and a higher overall semantic relatedness

So far, studies on VF performance including clustering and switching in individuals with ASD without intellectual impairment are rare and have reported partly contradictory findings (Begeer et al. 2014; Inokuchi and Kamio 2013; Spek et al. 2009; Turner 1999): corresponding analyses showed reduced word production in semantic and letter fluency (Spek et al. 2009; Turner 1999) with smaller (Turner 1999) or similar cluster sizes (Spek et al. 2009), specific semantic deficits (Inokuchi and Kamio 2013) as well as longer switches with unimpaired overall word production (Begeer et al. 2014) compared to individuals without ASD. All of these studies used a paradigm based on established semantic subcategories (Troyer et al. 1997) to define semantic clusters. Alternatively, temporal cluster analysis allows for an identification of clusters and switches independently from a-priori defined semantic subcategories by means of their temporal patterning using a curve fitting approach (Bousfield and Sedgewick 1944; Ehlen et al. 2016; Gruenewald and Lockhead 1980). To this end, consecutive words are plotted as a function of time such that ‘temporal clusters’ correspond to curve sections with a steeper slope than predicted by the formula’s graph, i.e. a faster production rate (see Fig. 1a).

In the current study, we combined a temporal cluster analysis with co-occurrence data from a corpus-driven database (Belica 2001) to estimate semantic relatedness within and outside of clusters. To this end, we assessed the co-occurrence value between each two consecutive words and connected the respective values to the word position within vs. outside a cluster for each participant. A high co-occurrence value indicates that two words typically occur in the same context and can thus be expected to be semantically related, whereas a low value should be indicative of low semantic relatedness (Smadja 1989). Higher values within than outside of clusters should therefore be expected. Furthermore, whereas co-occurrence data refer to a corpus, i.e. shared common knowledge, the words produced by the speaker during VF presumably reflect individual associations (cf. Murphy and Medin 1985). Individually low co-occurrence values within clusters could therefore be interpreted as atypical semantic associations.

From this perspective, the following parameters were deemed to be meaningful regarding semantic network organisation and executive functions: semantic relatedness (i.e. co-occurrence value) should provide an estimate of the typicality of associations, ‘cluster size’ (i.e. number of words per cluster) should represent the scope of densely connected words, ‘intracluster time’ (i.e. time between consecutive words within a cluster) the respective strength between established associations, the ‘number of switches’ should relate to the number of accessible items (either single words or clusters) and ‘switch duration’ (i.e. time between two words not belonging to the same cluster) to their accessibility (see Fig. 1b).

For individuals with ASD, hypoconnectivity within the semantic network could therefore be expected to either cause longer intracluster times as an expression of weaker interitem associations (see Fig. 1c) or reduced cluster sizes as an expression of a lower number of densely connected words (see Fig. 1d). Individual deviations of semantic associations (cf. Dunn et al. 1996), should be indicated by a lower semantic relatedness (i.e. lower co-occurrence value; see Fig. 1e) within clusters. Longer and fewer clusters could, on the other hand, be indicative of reduced set shifting leading to a longer attachment to the present cluster (see Fig. 1f; cf. Reverberi et al. 2006). Lastly, longer and fewer switches could hint at a slower access to new semantic fields (not depicted; cf. Troyer et al. 1997; Turner 1999).

In addition to semantic relatedness, orthographic relatedness was assessed between each two consecutive words within and outside of clusters. This was done under the premise that a bias towards a detail-focussed pattern processing (Huemer and Mann 2010; Nation 1999) could account for an advantage in orthographic processing among individuals with ASD without intellectual impairment (Huemer and Mann 2010; Minshew et al. 1994; Nation 1999; Saldaña et al. 2009). Finally, to test if the ASD group produces fewer socio-emotional and motor action words than the non-ASD group, we introduced the semantic categories ‘human features’ and ‘verbs’ along with a semantic control condition (i.e. ‘animals’). In addition to the above-named measures, both categories underwent an analysis of their semantic content (not performed for the other two conditions under the idea that they do not specifically convey socio-emotional or motor action related content).

### Summary of Study Hypothesis

The primary focus of the current study was to compare lexical network functions between adults with and without ASD without intellectual impairment by means of VF analysis. In this regard, we expected lower semantic relatedness within clusters as a marker for atypical semantic associations, longer intracluster times to indicate weaker interitem associations, and smaller clusters as a marker for a lower number of densely connected words for the ASD vs. non-ASD group. These differences were predicted to occur mainly in the three semantic task conditions as opposed to the letter fluency condition. Conversely, higher orthographic relatedness as a marker for advanced orthographic processing in the ASD vs. non-ASD group appeared feasible. Lastly, potential difficulties of the ASD group to produce words conveying socio-emotional or motor action related content were assessed by a semantic analysis of the task conditions ‘features’ and ‘verbs’.

## Methods

### Participants

Female and male adults with ASD (n = 32) as well as individuals without ASD (referred to as ‘non-ASD’; n = 39) were recruited for the current study, which was part of the Autect-Study (Cho et al. 2020). Exclusion criteria were IQ below 85 to ensure the comprehension of the instructions, current antipsychotic or anticonvulsant medication to eliminate potential neurological side effects, non-native German speakers, comorbid neurological disorders (i.e. demyelinating, neurodegenerative, or vascular disorders such as multiple sclerosis, Parkinson's disease, Huntington's disease, or Alzheimer's disease or a history of stroke), and age over 65 years to avoid possible confounding age-related neurodegeneration. In the non-ASD group, a history of any psychiatric disorder or Autism Spectrum Quotient (AQ Baron-Cohen et al. 2006;) above 32 points were exclusionary. According to their self-reported family histories, none of the non-ASD participants had a first-degree relative with ASD.

All participants with ASD were recruited via the ASD outpatient clinic of our university hospital. The non-ASD group was recruited from the PESA (Psychological Experimental Server Adlershof) database and via postings.

The ASD diagnostic process was performed by an expert in the field based on clinical interviews and scale assessments, encompassing a structured interview according to DSM-5 as well as the Autism Diagnostic Observation Schedule (ADOS, module 4 (Lord et al. 1989; Rühl et al. 2004) to quantify atypicalities in social and communicative behaviour by means of structured and semi-structured tasks. The Autism Diagnostic Interview-Revised (ADI-R; Lord et al. 1994; Bölte and Poustka 2005) was additionally conducted if a parent was available (n = 21). An ASD diagnosis was given if an individual fulfilled all required DSM-5 criteria. Participants from both groups were furthermore tested for tendency towards autistic-like traits using the AQ consisting of fifty questions (scores below 26 points indicate few or no autistic traits; scores above 25 are indicative, and scores above 32 are highly predictive of ASD). We used the “Wortschatztest” (WST; i.e. “vocabulary test”) (Schmidt and Metzler 1992) to assess the verbal intelligence quotient (VIQ) in all participants. The WST requires the recognition of 42 increasingly complex target words, each of which is presented in a list of five distractor pseudowords (e.g. “Sebtion–Pavisol–Arkusion–Epuktion–Savasor–Eruption”; Eruption being the target word). Test execution requires approximately 10 min. The test correlates highly with the Wechsler Adult Intelligence Scale, such that it can be used to estimate crystallized intelligence (Satzger et al. 2002).

All interviews and tests were carried out by specially trained psychologists and psychiatrists.

Participants from both groups did not differ significantly with respect to age, sex, or verbal IQ; as expected, a large difference was found regarding the AQ (see Table 1 for an overview).

**Table 1:** Overview participants: the table provides mean values and standard deviations (SD) from participants with Autism Spectrum Disorder (ASD) and without (non-ASD) regarding age, verbal intelligence quotient (VIQ), and the autism spectrum quotient (AQ; values < 25 are indicative of no autistic traits, values > 32 are highly predictive of ASD) as well as the distribution of female and male participants

	ASD		Non-ASD		*p* value	*F*_1,69_	Cohen’s *d*
	Mean	*SD*	Mean	*SD*			
Age (years)	37.063	10.692	34.974	7.768	.344	.906	− .223
Sex	f: 14; m: 18		f: 22; m: 17		2.880		
VIQ (points)	111.452	11.463	103.744	34.778	.241	.350	− .298
AQ (points)	37.290	5.751	14.314	6.173	< .001	242.758	− 3.851
ADOS (points)	9.188	3.247					

ADOS scores were not assessed in the non-ASD group. Group differences were generally tested using *t* tests except for the dichotomous parameter *sex* (chi-squared test). Cohen’s *d* was computed only for significant parameters

### Material

#### Verbal Fluency Tasks

All participants performed a Verbal Fluency (VF) test based on the German standard VF “Regensburger Wortfluessigkeitstest” (Aschenbrenner and Tucha Lange 2001). The test requires participants to produce as many words as possible pertaining to either a predefined semantic category (i.e. semantic fluency) or commencing with a given letter (i.e. letter fluency) within 120 s.

### Design

#### General

The dependent variables *total number of words*, *cluster size*, *intracluster*, *number of switches*, *switch duration*, *semantic relatedness*, *orthographic relatedness*, and *semantic category* (please see Procedures for details) were analysed by means of separate linear mixed models (LMMs) detailed below. Acknowledging the fundamental differences between semantic and letter based retrieval processes (Birn et al. 2010; Robinson et al. 2012; Vonberg et al. 2014) outlined in the Introduction, the analyses were performed separately for semantic and letter fluency task conditions (i.e. the three semantic task conditions were collapsed per LMM analysis; the letter fluency condition was analysed separately).

Due to processing speed-mediated effects of age on VF performance (Elgamal et al. 2011), subject outliers were assessed using Cook’s distances (*age* as independent; ‘number of words’ as dependent variable) separately for both groups prior to the main analyses. Values were considered as relevant if individual Cook’s distance exceeded three times the mean value and subjects were excluded if relevantly elevated values were detected in more than one task condition.

Data contribution was assessed using the Kolmogorov–Smirnov test. All statistical operations were carried out using the software IBM SPSS Version 25.

#### Speech Velocity

In advance of the main analyses, we compared average word lengths between both groups via t-test to control for group differences in speech velocity.

#### Verbal Fluency Performance, Semantic Fluency

Five separate LMMs were performed to evaluate effects of group and task condition on the five dependent parameters *total number of words*, *cluster size*, *intracluster time*, *number of switches*, and *switch duration* across all three semantic task conditions. For each LMM *group* and *task condition* served as main fixed factors. The variables *sex* and *age* were included as covariates.

#### Verbal Fluency Performance, Letter Fluency

The same five dependent variables were assessed in five separate linear mixed models each with the main fixed factor *group* and the covariates *sex* and *age*.

#### Semantic and Orthographic Relatedness, Semantic Fluency

To assess semantic relatedness within vs. outside of clusters across all three semantic task conditions, a LMM was applied using the dependent variable *semantic relatedness* and the main fixed factors *group*, *task condition*, and *word position within vs. outside of clusters*. *Sex* and *age* served as covariates.

*Orthographic relatedness* was assessed with the same approach in a separate LMM.

#### Semantic and Orthographic Relatedness, Letter Fluency

In the letter fluency condition, *semantic relatedness* served as dependent variable, *group* and *word position within vs. outside of clusters* as main fixed factors and *sex* and *age* as covariates. *Orthographic relatedness* was assessed similarly in a separate LMM.

#### Semantic Content

With respect to ‘human features’, we used a LMM with *number of words* as dependent variable and the main factors *semantic category* (i.e. ‘external features’, ‘human dispositions/feelings’, ‘other’) and *group*. The same model was applied regarding the semantic content of ‘verbs’ (i.e. ‘intentional physical actions’, ‘inactive states’, ‘mental actions’, and ‘non-action verbs’).

#### Error Rates

Kruskal–Wallis analyses for non-normally distributed data were used to evaluate group differences between the total number of errors (expressed as percentage value of the respective total number of words) per task condition.

#### Level of Significance and Effect Sizes

Altogether 16 LMMs were computed to assess the study outcome parameters. Therefore, Bonferroni correction yielded an adjusted level of significance of *p* < .003 (applicable to the main effects and interactions of each LMM). Regarding demographic and symptom related data (i.e. word length, age, verbal IQ, AQ, and sex) the adjusted level of significance was *p* < .01. The adjusted level of significance for Pearson’s correlation was *p* < .008. Since the use of Bonferroni procedures increases the probability of Type II errors, potentially informative effects could be missed (Nakagawa 2004). We therefore report *p* values obtained from the single statistical evaluations along with measures of effect sizes (cf. Wasserstein and Lazar 2016). Since there is no unequivocal effect size index for LMM (Lüdecke 2017), we used Cohen’s *d* (> .2: small; > .5: medium; > .8: large effect size; Cohen 1988b) for main fixed factors with two levels (i.e. *group* and *word position within vs. outside of clusters*) and eta squared (*η*^2^ > .01: small; > .06: medium; > .14: large effect size; Cohen 1988a) obtained from univariate ANOVAs for main fixed factors with more than two levels (i.e. *task condition* and *semantic category*), covariates, and non-normally distributed data (i.e. errors). Furthermore, when a LMM indicated an effect of group, correlations were carried out between the dependent variable and the AQ.

### Procedure

#### Verbal Fluency Task

Participants were instructed to avoid repetitions of whole words, word stems, and proper names. Semantic fluency comprised the three categories ‘*animals’*, ‘*human features’* and ‘*verbs’.* Whereas the first category served as control condition, the other two served to assess group differences specifically related to the socio-emotional or motor action related semantic content. For letter fluency, the common starting letter ‘r’ was chosen. The order of all four tests was randomised. Samples were digitally recorded (computer software Audacity 1.3.13-beta), annotated and analysed acoustically and visually using Praat software (version 6.0.40) by determining the starting and ending point of each uttered word (i.e. ‘word length’ in seconds with three decimal places). Metacomments (e.g. ‘I can´t come up with more words’) were not included in the analysis, while errors were assumed to be of informative value (cf. Troyer et al. 1997).

#### Verbal Fluency Performance

In order to evaluate clusters and switches, the individual data underwent a curve fitting process using the power function *n*(*t*) = *c*⋅[1 − (1 + *α · r · t*/*c*)^− 1/*α*^] established specifically for VF analysis (with *n*: number of words produced; *t*: time (in seconds); *c*: asymptote (in words); *α*: shape parameter (dimensionless); *r*: initial rate (in words/min) Ehlen et al. 2016;)*.* To this end, the starting point of each word was plotted as a function of time (word number on the ordinate; starting time in seconds on the abscissa) for each participant and condition. In accordance with the slope-difference algorithm (Gruenewald and Lockhead 1980), clusters were defined as two or more consecutive words produced at a faster rate than predicted by the individual graph, whereas switches were defined as slower transitions between two clusters (see Fig. 1a). Thus defining clusters as relative to the individual production curve, a person’s overall response speed does generally not impact on the distinction between clusters and switches.

We determined the *total number of words*, *cluster size* (i.e. number of words per cluster), *intracluster time* (i.e. average duration from word onset to next word onset within clusters), *number of switches*, and *switch duration* (i.e. average duration from word onset to next word onset of switches).

#### Semantic Relatedness

Semantic relatedness is generally assumed if two items typically occur in the same context, such that co-occurrence analyses of textual data have been established to assess the strength of semantic relatedness (Smadja 1989). In the current study semantic relatedness between each two consecutive words was evaluated using the corpus-driven co-occurrence database (CCDB) of the Leibniz Institute for the German Language (Belica 2001). The database includes information extracted from a corpus of about 2.2 billion running words forming a collection of co-occurrence profiles of about 222,000 different lemmas (Belica 1995). Semantic relatedness is expressed by a size between 0 (i.e. no relatedness) and 1 (identity) with an accuracy of 6 decimal places. For this purpose, all words were converted into their lemma form. If a word was not included in the database, it was substituted by the closest related word found in the CCDB (e.g. ‘brown bear’ instead of ‘grizzly bear’). In total, 133 out of 3604 words (3.690%) were replaced in the ASD group and 166 out of 4821 (3.443%; chi-squared test: *p* = .157) in the non-ASD group.

Based on the temporal cluster analysis, the co-occurrence value for each two consecutive words produced by each participant was then allocated to the individual word position as either belonging to a cluster or a switch. For further statistical analysis, the mean co-occurrence value within and outside of clusters was computed per person and task condition.

#### Orthographic Relatedness

All tasks were evaluated regarding orthographic relatedness. In individuals without ASD, orthographic relatedness has been shown to facilitate lexical retrieval in word recognition (e.g. Chéreau et al. 2007; Welcome and Trammel 2017) and production (e.g. Lupker 1982; Starreveld and La Heij 1996). Orthographic relatedness is typically assessed by the Levenshtein distance (Levenshtein 1966; Yarkoni et al. 2008) defining ‘distance’ by the number of letter alterations (i.e. insertions, deletions, or substitutions) needed to transform one word into another (e.g. transformation from ‘rope’ to ‘rules’: substitution of ‘o’ by ‘u’ + substitution of ‘p’ by ‘l’ + insertion of ‘s’ equals a Levenshtein distance of 3). Lower values of the dimensionless parameter thus indicate a stronger orthographic relatedness. In the current study, Levenshtein distances were computed between each two consecutive words to assess orthographic relatedness per condition and participant (please refer to the Appendix for the computation of the Levenshtein distance). For further statistical analysis, the mean Levenshtein distances within and outside of clusters was computed per person and task condition in a parallel manner as described above for semantic relatedness.

#### Semantic Content

We assessed the semantic content of the task conditions ‘human features’ and ‘verbs’. Human features were categorised according to the six semantic main-classes suggested by Baumann et al. (2018) (see Appendix Table 5). Special interest lay in the differentiation between adjectives describing ‘external features’ (e.g. ‘colour’, ‘sensory characteristics’) and ‘human dispositions/feelings’ (including ‘physical feeling’, ‘behaviour’, ‘mental state’). Acquisition of the latter follows that of more concrete external features during childhood and is thought to be related to the development of a theory of mind (Baumann et al. 2018). We therefore summarised Baumann's first four main-classes (i.e. *age*, ‘colour’, ‘judgments’, and ‘sensory characteristics’) as ‘external features’ and maintained the fifth (i.e. ‘human dispositions/feelings’) as well as the unspecific sixth (i.e. ‘other’) main-class.

The semantic analysis of the 'verbs' condition served to assess the production of movement and non-movement related action words. In general, most verbs are defined as action verbs, except for auxiliary, modal, and copula verbs. A variety of systems have proposed a sub-classification of action verbs by their semantic content, commonly dividing them into ‘mental actions’ (e.g. ‘thinking’, ‘feeling’, and ‘sensing’) and ‘physical actions’ with a sub-sub-classification into ‘intentional physical actions’, ‘non-intentional processes’, and ‘states’ (Bär 2015; Hentschel and Weydt 2013; Stanford NLP Group 2019) (see Appendix Table 6). On this basis, we differentiated between ‘intentional physical actions’, ‘inactive states’ (i.e. sum of ‘non-intentional processes’ and ‘states’), ‘mental actions’, and ‘non-action verbs’ (i.e. sum of auxiliary, modal, and copula verbs) for further group comparisons. Absolute values were used for all analyses of semantic content.

## Results

### Speech Velocity

Word length did not differ significantly between the two groups (ASD: .954 ± .493 s; non-ASD: .869 ± .119 s; *p* = .432; *F*_1,43_ = 3.039; Cohen’s *d* = .239). No subject outliers were detected.

### Verbal Fluency Performance

#### Semantic Fluency

The five LMMs performed to assess VF performance across the three semantic task conditions suggested a lower *total number of words*, smaller *cluster sizes*, and longer *switch durations* among individuals with ASD compared to the non-ASD group. Effect size of *group* was moderate for *cluster size* and small for *total number of words* and *switch duration.* However, none of the *p* values, reached the adjusted level of significance (see Table 2A). No group differences were indicated regarding *intracluster time* and *number of switches*.

**Table 2:** Verbal fluency performance: the tables show verbal fluency performance as mean values and standard deviations (SD) of in (A) the three semantic fluency task conditions and (B) the letter fluency task condition in participants with Autism Spectrum Disorder (ASD) and without (non-ASD)

(A) Semantic tasks							
	ASD		Non-ASD		*p* value	*F*_1__,207_	Cohen’s *d*
	Mean	SD	Mean	SD			
Total number of words	31.021	9.504	34.607	7.808	.015	6.011	.412
Intracluster time (s)	2.400	1.140	2.160	.686	.389	.744	− .256
Cluster size (words)	3.481	.568	3.772	.526	.007	7.476	.532
Number of switches	11.281	3.395	12.051	2.962	.189	1.736	.242
Switch duration (s)	6.815	2.166	6.045	1.344	.039	4.331	− .427

(B) Letter fluency							
	ASD		Non-ASD		*p* value	*F*_1,67_	Cohen’s *d*
	Mean	SD	Mean	SD			
Total number of words	19.563	7.094	19.795	5.850	.598	.281	.036
Intracluster time (s)	4.082	1.710	3.924	1.885	.606	.268	− .088
Cluster size (words)	3.712	1.900	3.567	1.011	.790	.072	− .095
Number of switches	6.875	3.210	6.897	2.521	.768	.088	.008
Switch duration (s)	9.542	4.586	9.726	4.574	.884	.022	.040

All group comparisons were computed using linear mixed models (adjusted level of significance *p* < .003)

Regarding effects of *task condition* across both groups, the highest *number of words* and *switches* together with shortest *intracluster times* and *switch durations* was found in the condition ‘animals’, followed by ‘verbs’ and ‘human features’. Except for *cluster size* all *p *values reached the adjusted level of significance with moderate to large effect sizes (see Appendix Table 7). The models suggested no significant interactions between *group* and *task condition* (see Appendix Table Table 8). Despite generally large effect sizes of *age,* no significant effects of the covariates were indicated (see Appendix Table 9).

Pearson’s correlation suggested a tendency towards a weak negative relationship between *AQ* and the *total number of words* in the ASD group without reaching the level of significance (ASD: *r* = − .335, *p* = .065; non-ASD: *r* = − .181, *p* = .298) and no significant relationship between *AQ* and *cluster size* (ASD: *r* = − .202, *p* = .276; non-ASD: *r* = − .045, *p* = .798) or *switch duration* (ASD: *r* = .107, *p* = .568; non-ASD: *r* = − .067, *p* = .702).

#### Letter Fluency

Regarding the letter fluency task, the LMMs suggested no significant effects of *group* on the same measures as above along with very low effect sizes (see Table 2B).

The models suggested no significant effects of the covariates on the dependent variables (see Appendix Table 10).

### Semantic Relatedness

#### Semantic Fluency

The LMM including all three semantic task conditions to analyse *semantic relatedness* suggested no significant effect of *group* along with a very small effect size (ASD: .154 ± .086; non-ASD: .159 ± .091; *p* = .335; *F*_1,411_ = .932; Cohen’s *d* = .056).

**Fig. 2:**
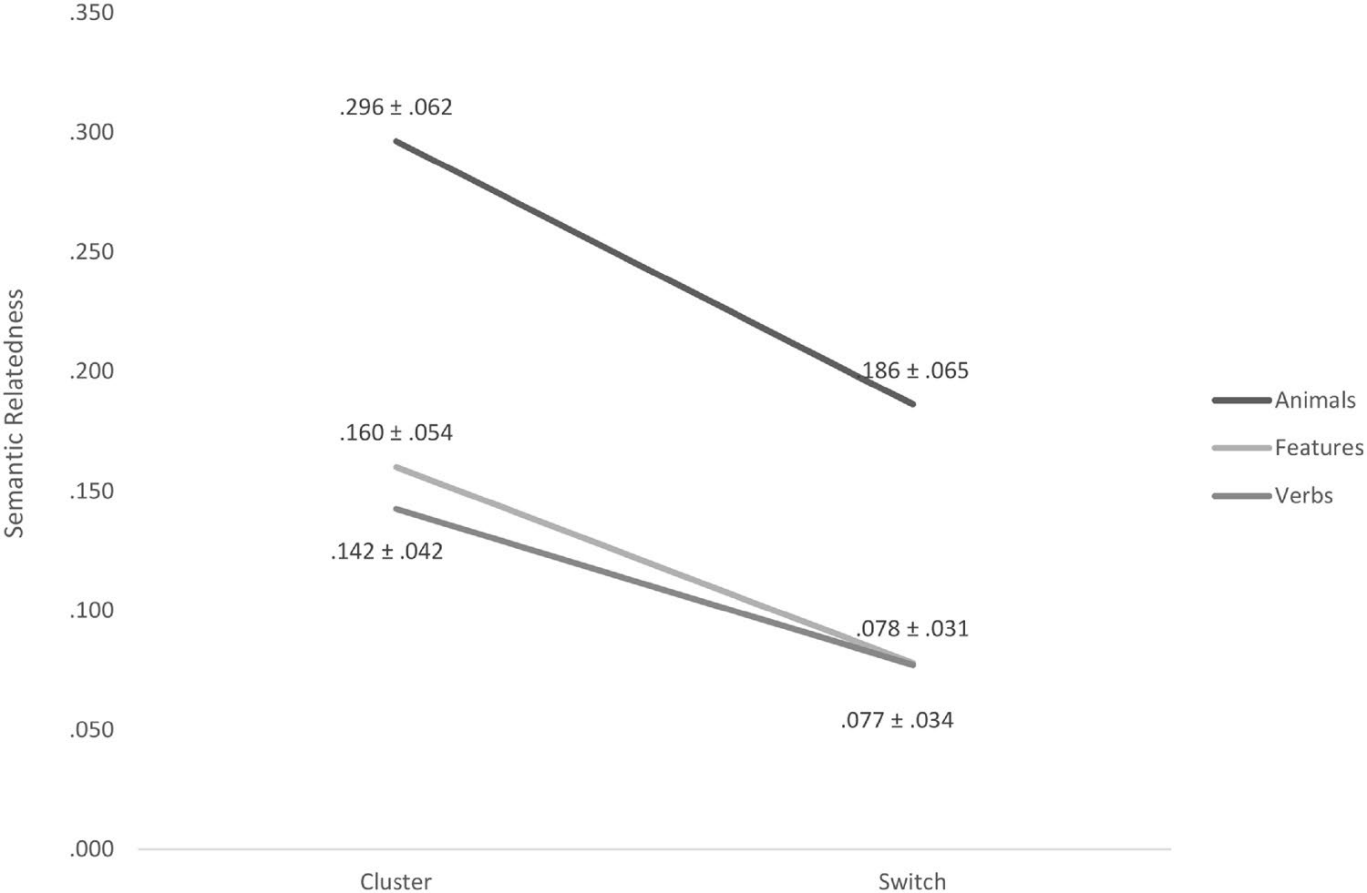
Semantic Relatedness. Regarding semantic relatedness across the three semantic task, there were highly significant effects of *task condition* (i.e. ‘animals’, ‘features’, ‘verbs’) and *word position within vs. outside of clusters* as well as an interaction between these two factors (each p < .001) but no differences between participant groups. Values are displayed as mean values from both participant groups for each of the three semantic tasks. The ordinate indicates semantic relatedness (ranging from 0 to 1)

The model suggested a significant effect of *word position within vs. outside of clusters* with higher relatedness within than outside of clusters with a *p *value below the adjusted level of significance and a large effect size (within cluster: .201 ± .087; outside cluster: .113 ± .066; *p* < .001*; F*_1,411_ = 336.743; Cohen’s *d* = − 1.140) across both groups (interactions between *group* and *word position within vs. outside of clusters*: *p* = .325; *F*_1,411_ = .970). Also the effect of *task condition* appeared significant with highest relatedness between ‘animals’ and lowest between ‘verbs’ with a *p* value below the adjusted level of significance and a large effect size (‘animals’: .241 ± .082; ‘human features’: .119 ± .061; ‘verbs’: .110 ± .050; *p* < .001; *F*_2,411_ = 314.318; *η*^2^ = .453) across both groups (interactions between *group* and *task condition*: *p* = .409; *F*_2,411_ = .897). The model furthermore suggested a significant interaction between *word position within vs. outside of clusters* and ‘task conditions’ (*p* < .001; *F*_2,411_ = 8.490) across both groups (see Fig. 2). Effects of the covariates were not suggested to be significant (*age*: *p* = .503; *η*^2^ = .344; *F*_1,411_ = .450; *sex*: *p* = .766; *F*_1,411_ = .089; *η*^2^ = .002).

#### Letter Fluency

With respect to letter fluency, *semantic relatedness* was comparably low with high standard deviations. Values were almost identical in both participant groups (ASD: .030 ± .032; non-ASD: .030 ± .037; *p* = .923; *F*_1,136_ = .009; Cohen’s *d* < .001). Relatedness was slightly higher within than outside of clusters (within cluster: .038 ± .036; outside cluster: .022 ± .032; *p* = .010;* F*_1,136_ = 6.853*;* Cohen’s *d* = − .470) without reaching the adjusted level of significance. The LMM suggested no significant interaction between the factors *group* and *word position within vs. outside of clusters* (*p* = .239; *F*_1,136_ = 1.396) and no significant effects of the covariates (*age*: *p* = .795; *F*_1,136_ = .068; *η*^2^ = .167; *sex*: *p* = .470; *F*_1,136_ = .524; *η*^2^ = .007).

### Orthographic Relatedness

#### Semantic Fluency

The LMM including all three semantic task conditions to analyse *orthographic relatedness* suggested no significant effect of *group* along with a very small effect size (Levenshtein distance ASD: 6.652 ± 1.420; non-ASD: 6.576 ± 1.340; *p* = .393; *F*_1,409_ = .731; Cohen’s *d* = − .055). It suggested a significant, moderate effect of *word position within vs. outside of clusters* with higher relatedness within than outside of clusters (Levenshtein distance within cluster: 6.281 ± 1.251; outside cluster: 6.938 ± 1.470; *p* =  < .001; *F*_1,409_ = 65.518; Cohen’s *d* = .481) across both groups (interactions between *group* and *word position within vs. outside of clusters*: *p* = .150; *F*_1,409_ = 2.082). The model suggested a large effect of *task condition* with highest relatedness between ‘verbs’ and lowest between ‘human features’ (Levenshtein distance ‘verbs’: 5.343 ± .698; ‘animals’: 6.497 ± .767; ‘human features’: 8.019 ± 1.118; *p* < .001, *F*_2,409_ = 384.175; *η*^2^ = .609) across both groups (interactions between *group* and *task condition*: *p* = .277; *F*_2,409_ = 1.288). Lastly, the LMM suggested a small effect of *sex* across both groups and all three task conditions (Levenshtein distance female: 6.634 ± 1.284; male: 6.448 ± 1.251; *p* = .014; *F*_1,409_ = 6.109; *η*^2^ = .032), which did not reach the adjusted level of significance. No significant effect of *age* was suggested (*p* = .488; *F*_1,409_ = .482; *η*^2^ = .341).

#### Letter Fluency

Regarding the letter fluency task, the LMM suggested that *orthographic relatedness* did not differ significantly between the groups (Levenshtein distance ASD: 5.578 ± 1.357; non-ASD: 5.650 ± 1.312; *p* = .759; *F*_1,136_ = .094; Cohen’s *d* = .054) but within vs. outside of clusters (Levenshtein distance within cluster: 5.269 ± 1.186; outside cluster: 5.966 ± 1.379; *p* = .002; F_1,136_ = 10.175; Cohen’s *d* = .543). The model indicated no significant interactions between *group* and *word position within vs. outside of clusters* (*p* = .898; *F*_1,136_ = .016) and no significant effects of *age* (*p* = .686; *F*_1,136_ = .164; *η*^2^ = .376) or *sex* (*p* = .640; *F*_1,136_ = .219; *η*^2^ = .003).

### Semantic Content

The LMM assessing the semantic content of the 'human features' condition suggested no significant effect of *group* (*p* = .201; *F*_1,214_ = 1.584; Cohen’s *d* = .125) but a significant and large effect of *semantic category* (‘human dispositions/feelings’: 16.417 ± 9.110 words; ‘external features’: 7.306 ± 6.324 words; ‘other’: 1.222 ± 1.606 words; *p* < .001, *F*_2,100_ = 21.709; *η*^2^ = .541) without a significant interaction between *group* and *semantic category* (*p* = .439; *F*_2,214_ = .827).

With respect to the semantic content of the ‘verbs’ condition, the LMM suggested no significant effect of *group* (*p* = .204; *F*_1,141_ = 1.626; Cohen’s *d* = .159) but a significant and large effect of *semantic category* (*p* < .001, *F*_3,274_ = 421.695; *η*^2^ = .818). The model suggested an interaction between *group* and *semantic category* (*p* = .022; *F*_3,274_ = 3.246), however not reaching the adjusted level of significance. Post-hoc t-tests showed a tendency towards fewer ‘intentional physical actions’ in the ASD vs. non-ASD group (see Table 3), without reaching the adjusted level of significance.

**Table 3:** Semantic content, verbs: given a significant interaction between *group* and *semantic category* of verbs, post-hoc tests were performed to assess content specific group differences regarding the semantic content of verbs (Mann–Whitney-test for ‘non-action verbs’, otherwise *t* tests)

	ASD		Non-ASD		*p* value	*F*_1,69_	Cohen’s *d*
	Mean	SD	Mean	SD			
Intentional physical actions	22.344	7.677	25.667	6.768	.057	3.753	.459
Inactive states	5.969	4.139	5.590	2.603	.64	.221	− .110
Mental actions	5.344	3.298	4.795	3.088	.472	.522	− .172
Non-action verbs	0.188	0.64	0.128	0.409	.955	n.a.	− .111

*n.a*. not applicable

No significant group effects were found. Values are given as absolute number of words produced per semantic category

### Error Rates

Kruskal–Wallis analyses suggested no group differences in error rates, although large standard deviations in some conditions may have obscured effects (see Table 4). Specifically, in the condition, 'human features', the error rate was more than three times as high in ASD compared to non-ASD.

**Table 4:** Errors: the total number of errors is given as percentage of the total number of words per task condition and group

Errors, total (%)	ASD		Non-ASD		*p* value	*η*^2^
	Mean	*SD*	Mean	*SD*		
Semantic						
Animals	5.548	7.188	3.809	3.506	.791	.025
Features	7.732	17.788	3.997	5.213	.459	.022
Verbs	2.483	4.146	2.335	4.733	.751	< .001
Letter						
r-Words	5.408	7.030	4.453	5.696	.537	.006

Group comparisons were performed by Kruskal–Wallis analyses

## Discussion

The current study explored semantic and letter fluency in individuals with ASD without intellectual impairment in an attempt to characterise underlying lexical network organisation. Therefore, the primary focus was on differences between the ASD and non-ASD group regarding VF performance, semantic and orthographic relatedness as well as content specific group differences. Analyses of the single VF parameters suggested moderately smaller clusters among individuals with ASD compared to non-ASD along with slightly longer switches and hence a slightly lower number of words in all three semantic task conditions. However, none of these group differences reached the level of significance if corrected for multiple comparisons. There appeared to be no substantial group difference regarding intracluster time or the number of switches. Conversely, in the letter fluency condition, our analyses suggested no relevant group differences regarding clusters, switches, or overall performance. With respect to semantic and orthographic relatedness between consecutive words, no group differences were identified. Yet, both semantic and orthographic relatedness appeared to be significantly stronger within clusters compared to switches. Content analyses of the ‘human features’ and ‘verbs’ condition indicated no group-specific differences in the production of socio-emotional or motor action related words. There was no indication of group differences in error rates.

However, comparably small effect sizes of *group* for each variable point towards a low statistical power so that possible group differences may not have been identified. Application of multiple LMM and the respective adjustment of the level of significance furthermore increased the probability of Type II errors. Therefore, effects which were indicated as significant at the uncorrected level of significance could still be of potential interest and shall therefore be discussed in the theoretical framework of the lexical network, executive functions, and content specific performance.

### Lexical Network

Smaller clusters leading to a lower overall word production only in the semantic task conditions appeared to constitute the most relevant difference between the ASD group and non-ASD in the current study. Reduced word production in semantic fluency tasks among individuals with ASD without intellectual impairment has been described in two earlier studies indicating either pronounced deficits in semantic vs. letter fluency task conditions (Spek et al. 2009) or deficits exclusively in semantic tasks (Inokuchi and Kamio 2013). However, the same authors reported no group differences regarding clusters and switches. Reduced overall word production was also reported by Turner (1999), who furthermore found smaller semantic clusters in both semantic and letter fluency tasks in individuals with ASD compared to non-ASD. This was interpreted as a limited capacity of individuals with ASD to profit from semantic and phonemic relatedness (Turner 1999). Due to basic methodological differences between the use of predefined semantic clusters in the above studies and temporal clusters in the current one, comparability is limited. However, in contrast to Turner (1999) the present findings provided no indication of group differences regarding the use of semantic or phonemic relatedness.

In the framework of the proposed network model, comparably small clusters among individuals with ASD could be indicative of a relatively small scope of closely associated words within the semantic network. Concurrently, in the present study, intracluster time and the estimate of semantic relatedness were largely similar in both groups and thus indicative of similar strength and typicality of established semantic associations. This interpretation appears consistent with two predictions of the WCC theory (Happé and Frith 2006): Firstly, subordination of single items to a higher order category seemed particularly vulnerable to the proposed bias towards detail-focussed processing. Secondly, lower level integration of featural aspects appeared largely unaffected.

WCC has been closely linked to functional hypconnecticvity of neural networks in ASD (Just et al. 2004). Regarding the language network, reduced functional and structural connectivity of various language areas has been associated with differences in language production (Knaus et al. 2010; Verly et al. 2014), comprehension (Goch et al. 2013; Just et al. 2004), and language development (Catani et al. 2016; Naigles et al. 2017) in ASD. A smaller number of closely connected words could thus be rooted in weak connections within areas binding semantic information. In this regard, a particular role for the connection of featural aspects to achieve higher-order generalization (Patterson et al. 2007) and for semantically-driven spoken word production (Mirman et al. 2015) has been ascribed to the anterior temporal lobe. It would therefore appear intriguing to relate behavioural data from temporal cluster analysis to connectivity measures of language specific sub-systems to challenge the here proposed interpretations.

The current study did not include an analysis of typicality or word frequency effects. This appears important to mention because typicality effects could have group-specific effects on VF measures. Word typicality refers to the observation that particular items which appear representative for a given category or share a high number of features with related items are more likely to be produced by a speaker (Rosch and Mervis 1975). Theoretical considerations (Murphy and Medin 1985) and empirical studies embracing, e.g. individual expertise (Bailenson et al. 2002) and socio-cultural background (Burnett et al. 2005) have highlighted that apart from item-inherent features, the speakers’ individual experiences and mindsets shape and organise their mental concepts. Considering the high prevalence (Klin et al. 2007) and the intensity (Anthony et al. 2013) of circumscribed interests among children with ASD without intellectual impairment, relevant differences in the organisation of mental concepts appear plausible. Moreover, differences in categorisation in early childhood have been proposed to contribute, e.g. to difficulties in social cognition and communication in individuals with ASD (Gastgeb et al. 2006). Gastgeb et al. (2006) showed specifically slower reactions to atypical exemplars of a category among individuals with ASD without intellectual impairment compared to non-ASD (Gastgeb et al. 2006). The authors argued that a bias towards feature-based rather than a holistic processing could entail slower access to the semantic information needed for the categorisation of atypical exemplars. In reverse, slower semantic access to atypical items could have caused smaller temporal clusters in the ASD group of the present study. Assessing group-specific typicality effects as well as corpus-based estimates of words frequencies could therefore be of value in future studies to characterise in how far personal learning histories shape individual semantic categories.

Of note, Turner (1999) reported even smaller clusters among participants with learning disability (both ASD and non-ASD) than among individuals with ASD without intellectual impairment. This could be interpreted in a way that in addition to ASD-specific effects on cluster size, differences in individual learning skills can also affect cluster formation in diverse participant groups.

As to letter fluency, our present results suggested no group differences in clustering, switching, or overall performance. Although the absence of group differences in this study might be due to low power, this could be in line with the presumption that detail-focussed processing may support orthographic word access strategies in ASD. In a similar sense, studies on reading skills (Nation et al. 2006) and lexical processing (Ferman and Bar-On 2017; Huemer and Mann 2010; Saldaña et al. 2009) in school children and adolescents with ASD have indicated a marked heterogeneity of reading ability with a typical discrepancy between impaired comprehension from semantic context and relatively good orthographical decoding (Huemer and Mann 2010; Nation 1999; Saldaña et al. 2009). These findings seem to corroborate a pattern of intact procedural orthographic abilities but comparably low comprehension skills described in adults with ASD without intellectual impairment (Minshew et al. 1994) and have been associated with difficulties integrating information in context (Huemer and Mann 2010; Nation 1999) as postulated by the WCC theory (Frith 1989). The way in which orthographic processing is involved in lexical decoding and word production is, however, still a matter of debate (Lupker 1982; Starreveld and La Heij 1996; Peleg et al. 2016; Seidenberg and McClelland 1989; Alario et al. 2007; Perret and Laganaro 2012): in individuals without ASD, orthographic facilitation has been described in picture-naming tasks (Lupker 1982; Starreveld and La Heij 1996) and direct connections with conceptual, semantic, and phonemic processing stages have been modelled (Peleg et al. 2016; Seidenberg and McClelland 1989) as well as refuted (Alario et al. 2007; Perret and Laganaro 2012) by experimental word production studies. The current findings may thus indicate a relative advantage in orthographic processing compared to semantic processing during verbal fluency tasks. This is in keeping with a predominance of semantic vs. letter fluency deficits in earlier studies (Inokuchi and Kamio 2013; Spek et al. 2009; Turner 1999). However, the assessment of orthographic relatedness in the present study did not indicate a superior use of orthographic strategies in the ASD vs. non-ASD group. On a side note, male participants across both groups showed a tendency towards higher orthographic relatedness in semantic tasks than female participants. Not reaching the adjusted level of significance and considering the small effect size, a higher statistical power could possibly have revealed group differences. It thus remains speculative if orthographic relationship may have served word retrieval specifically in males. But keeping in mind the “extreme male brain theory of autism” (Baron-Cohen 2002), a possible relationship between orthographic access strategies and sex could be of interest in future studies.

### Executive Function

The number of switches did not largely differ between groups. In the context of frontal lesions, fewer switches have been proposed as indicative of impaired set shifting (Reverberi et al. 2006). In view of a proposed impairment in cognitive flexibility in individuals with ASD (Kleinhans et al. 2005; Ozonoff et al. 2007), a reduced number of switches could have been expected. This notion also appears supported by a finding by Turner (1999) who showed an impaired ability to generate novel ideas in non-verbal fluency (i.e. ideational and design fluency) among able individuals with ASD without intellectual impairment. The present results, however, do not provide evidence for a corresponding dysfunction. Similarly, Spek et al. (2009) reported no difference in the number of switches. This should, however, not preclude the possibility of enhanced task demands inducing detectable difficulties in cognitive flexibility. In fact, fewer switches and a trend towards larger semantic clusters in the VF task condition ‘animals’ have been reported in children and adolescents with ASD without intellectual impairment (Begeer et al. 2010). This may have originated from relatively higher task demands for children compared to adults.

That said, we found switches to be slightly longer among participants with ASD compared to participants without ASD (not significant if corrected for multiple comparisons, likely due to low power). This could be interpreted in the sense of a proposed connectivity weakness of frontal areas (Catani et al. 2016; Courchesne and Pierce 2005) involved in accessing the semantic knowledge.

Furthermore, no group differences were indicated regarding error rates in the present study. Although the absence of this effect could be due to low power, it is in keeping with studies portrayed above reporting normal error rates in participants with ASD without intellectual impairment (Begeer et al. 2014; Dunn et al. 1996; Mottron et al. 2001; Turner 1999). In this context, an effect of IQ on executive function highlighted by Ozonoff et al. (2007) appears relevant, considering that in the present study, groups were matched for VIQ ranges. Lastly, the role of executive functions for VF performance has been challenged by a factor-analytic study determining a main impact of language functions on VF (Whiteside et al. 2016).

### Content Specific Performance

In regard to content related group differences, we assessed semantic subcategories of ‘human features’ and ‘verbs’. Against our hypothesis, groups did not differ with respect to the production of external human features vs. human dispositions/feelings (again, low power may have concealed possible group difference). Considering, a preference for factual knowledge (Klin et al. 2007) and sensory oriented interest areas (Anthony et al. 2013), a bias towards the production of external human features in the ASD group would have appeared feasible. A specific disadvantage in the production of words relating to social cognition had furthermore been proposed by Spek et al. (2009) who reported a particularly low word production in the VF category ‘professions’ in individuals with ASD without intellectual impairment (Spek et al. 2009). This hypothesis is, however, not supported by the present findings which rather appear in line with other studies describing intact usage of emotional words (Helen Tager-Flusberg and Sullivan 1995) as well as mental state and cognition terms during conversation in individuals with ASD (Bang et al. 2013). Corresponding findings may seem at odds with the typical social communicational difficulties, reports on fewer mental-state references (Begeer et al. 2010; Brown et al. 2012), and a suspected hypoconnectivity within the socio-emotional brain network (Ameis et al. 2011). This gap could, however, be explained by the observation of an unhindered production of, but impaired causal attribution of emotional terms in ASD (Capps et al. 2000; Losh and Capps 2006) and a reduced frequency of using emotional terms with increasing social complexity in ASD (Teh et al. 2018). The current results thus support the notion that socio-emotional terms are not specifically underrepresented if addressed independently from complex social situations.

With respect to the ‘verbs’ condition, we found a tendency towards fewer ‘intentional physical actions’ in the ASD group compared to non-ASD, which failed to reach statistical significance (possibly due to low power). An attenuation of motor-action verb production in individuals with ASD compared to non-ASD appeared plausible in the context of ‘embodied semantics’ which conceptualises the importance of sensorimotor engagement for multimodal language processing (e.g. Damasio et al. 1994; Kiefer and Pulvermüller 2012; Pulvermüller 1999). Thus, disorders of the motor system have been discussed as impediments to the processing of motor related language, e.g. verb production (Neininger and Pulvermüller 2001 cf. Aravena et al. 2014). Among individuals with ASD, motor dysfunctions—traditionally described as clumsiness (Asperger 1944)—including problems with posture, smoothness, and coordination (Rinehart et al. 2006) as well as disturbed gait (Jansiewicz et al. 2006; Rinehart et al. 2006), balance, and rhythmicity (Jansiewicz et al. 2006) are robustly found (for a meta-analysis see Fournier et al. 2010). Specific deficits in the VF conditions ‘sports’ and ‘action fluency’ reported by Inokuchi and Kamio (2013) seem to support this notion. To further explore embodied semantics and respective impairments in individuals with ASD, a stratification by motor symptoms may be considered in future studies.

### Study Design

From a conceptual point of view, it appears of interest that clusters solely defined by temporal patterning showed stronger semantic and orthographic relatedness than switches across all task conditions. This provides support for the theoretical background assuming strong connections between closely related words (both semantic and orthographic) to enable fast lexical access. To our knowledge, this is the first study combining VF analysis via curve fitting with an evaluation of semantic relatedness based on corpus-driven data. Since the present type of analysis allows for an objective evaluation of VF tasks irrespective of a-priori knowledge about semantic subcategories, it may be of value also for future studies.

### Limitations

Upon including a large number of statistical comparisons, none of the group effects, which were found in single LMMs reached the adjusted level of significance. The main findings therefore have to be interpreted with caution. Another critical point are low effect sizes regarding group effects across all dependent variables. These values suggest that the non-significant results are more likely due to low power of the current study and do not necessarily indicate the absence of group effects. Future studies could avoid this by using larger sample sizes. Furthermore, the assessment of only one letter fluency condition as opposed to three semantic conditions must also be critically remarked. This reduced the comparability and led to a large number of statistical comparisons.

## Conclusion

Adults with ASD without intellectual impairment appeared to produce smaller lexical clusters specifically in semantic fluency tasks compared to non-ASD. At the same time, typicality and strength of semantic associations did not seem to differ between groups. Taken together, the findings appear compatible with the ideas put forward in the WCC theory in that they could reflect a comparably weak subordination of single items to a higher order category, while integration of the single aspects of each item seemed unaffected. Moreover, orthographic retrieval strategies may have compensated for semantic network shortcomings. No indication was given for differences in the production of words conveying socio-emotional or motor action related content.

## Appendix 1

See Tables 5, 6, 7, 8, 9, and 10.

**Table 5:** Words produced in the category ‘human features’ were categorised semantically as proposed by Baumann et al. (2018; own translation given in the table)

	Main classes	Sub-classes	Example
1	Age		Old, new
2	Colour		Red
3	Appraisal		Important, bad
4_a	Sensory characteristics	Texture	Rough
4_b		Shape	Round
4_c		Dimension	Large
4_d		Consistency	Soft
4_e		Functionality	Broken
4_f		Purity	Dirty
4_g		Sensory impression	Hearty
4_h		Velocity	Fast
4_i		Temperature	Cold
4_j		Appearance	Dark
4_k		Palatability	Ripe
5_a	Human dispositions/feelings	Physical feeling	Hungry, sick
5_b		Behaviour	Decent, lazy
5_c		Mental State	Sad, clever
6_a	Other	Conformity	Unfit
6_b		Time	Early
6_c		Quantity	Rare, rich, full
6_d		Other	

For further analysis, we summarised the main-classes 1–4 as ‘external features’ and maintained the main-classes 5 and 6

**Table 6:** Categorisation of verbs was conducted based on common classification systems (Bär 2015; Hentschel and Weydt 2013; Stanford NLP Group 2019)

Category	Main classes	Sub-classes	Example
Action verbs	Physical actions	Intentional physical actions^a^	Run, throw, give, beat (usually used with object)
		Non-intentional Processes^b^	Fall, grow, die, decay
		States^b^	stand, lie, sit, stay
	Mental actions/sensing	Thinking^c^	Think, contemplate
		Wanting^c^	Like, hate, want, need
		Sensing^c^	See, smell, hear, taste
Non-action verbs	Copula verbs^d^		Have, be, appear, seem, smell, taste
	Auxiliary verbs^d^		Have, be
	Modal verbs^d^		Need, may, want, can, must, will

For further group comparisons, words were grouped as follows: ^a^‘intentional physical actions’; ^b^‘inactive states’; ^c^‘mental actions’; ^d^‘non-action verbs’

**Table 7:** Linear mixed models suggested significant effects of task condition for the three semantic task conditions (i.e. ‘animals’, ‘human features’, ‘verbs’) regarding verbal fluency parameters

	Animals		Features		Verbs		*p* value	*F*_2,207_	*η*^2^
	Mean	SD	Mean	SD	Mean	SD			
Total number of words	37.099	9.846	26.620	9.607	35.254	10.410	< .001	22.562	.176
Intracluster time (s)	1.901	1.152	2.757	1.558	2.096	.645	< .001	9.666	.080
Cluster size (words)	3.711	.627	3.446	.907	3.766	.824	.036	3.370	.030
Number of switches	13.070	3.603	9.451	3.949	12.592	4.251	< .001	17.917	.144
Switch duration (s)	5.672	1.685	7.658	2.793	5.827	2.021	< .001	15.200	.125

Generally, the largest number of words along with shortest intracluster times, most switches and shortest switch duration was found in the category ‘animals’ followed by ‘verbs’, and lastly ‘human features’

**Table 8:** Linear mixed models performed to assess VF performance parameters across all three semantic task conditions and both participant groups indicated no significant interactions between *group* and *task condition*

Interaction *group*task condition*	*p*	*F*_2,205_
Total number of words	.733	.311
Intracluster time (s)	.541	.616
Cluster size (words)	.361	1.025
Number of switches	.791	.235
Switch duration (s)	.749	.289

**Table 9:** Across all three semantic task conditions and both participant groups, linear mixed models indicated no significant effects of the covariates *age* and *sex*, although effect sizes of *age* were generally large

	Age			Sex		
	*p*	*F*_1,205_	*η*^2^	*p*	*F*_1,205_	*η*^2^
Total number of words	.082	3.058	.352	.733	.117	< .001
Intracluster time (s)	.176	1.846	.324	.823	.050	.002
Cluster size (words)	.914	.012	.327	.550	.359	.001
Number of switches	.092	2.863	.323	.403	.702	.006
Switch duration (s)	.308	.775	.272	.757	.096	.001

**Table 10:** For the letter fluency condition, linear mixed models indicated no significant effects of the covariates *age* and *sex* across both participant groups

	Age			Sex		
	*p*	*F*_1,67_	*η*^2^	*p*	*F*_1,67_	*η*^2^
Total number of words	.086	3.038	.338	.158	2.04	.019
Intracluster time (s)	.433	.623	.214	.575	.317	.002
Cluster size (words)	.734	.117	.237	.167	1.951	.033
Number of switches	.340	.923	.317	.227	1.488	.017
Switch duration (s)	.169	1.930	.282	.223	1.51	.016

## Computation of the Levenshtein Distance

The Levenshtein distance between two strings $a, b$ (of length $|a|$ and $|b|$ respectively) is given by ${\mathrm{l}\mathrm{e}\mathrm{v}}_{a,b}\left(|a|,|\mathrm{b}|\right)$ where

$${\text{lev}}_{a,b} \left( {i,j} \right) = \left\{ {\begin{array}{*{20}c} {\max \left( {i,j} \right)} & {{\text{if}}\min \left( {i,{ }j} \right) = 0,} \\ {{\min}\left\{ {\begin{array}{*{20}l} {{\text{lev}}_{a,b} \left( {i - 1, j} \right) + 1} \hfill \\ {{\text{lev}}_{a,b} \left( {i, j - 1} \right) + 1} \hfill \\ {{\text{lev}}_{a,b} \left( {i - 1, j - 1} \right) + 1_{{\left( {a_{i} \ne b_{j} } \right)}} } \hfill \\ \end{array} } \right.} & {{\text{otherwise}}.} \\ \end{array} } \right.$$

where ${1}_{\left({a}_{i}\ne {b}_{j}\right)}$ is the indicator function equal to 0 when ${a}_{i}={b}_{j}$ and equal to 1 otherwise, and ${\mathrm{l}\mathrm{e}\mathrm{v}}_{a,b}\left(i,j\right)$ is the distance between the first $i$ characters of $a$ and the first $j$ characters of $b$. $i$ and $j$ are 1-based indices.

To calculate the value of ${\mathrm{l}\mathrm{e}\mathrm{v}}_{{{word}}1, {{word}}2}$ for each pair of consecutive words $[word1,word2]$ in the VF tests, we used the following *perl* implementation of the Levenshtein distance:The calculated values of $\text{lev}_{{{word}}1, {{word}}2}$ were integers between 0 and $\text{max}{(\vert{word1}\vert, \vert{word2}\vert)}$
